# The Developmental Implications of the Use of Reproductive Technologies for Transgender People: A Comparative Cross-Section Protocol

**DOI:** 10.3389/fpsyt.2020.00243

**Published:** 2020-04-01

**Authors:** Grégor Mamou, Chrystelle Lagrange, Nicolas Mendes, Joy Wielart, Fanny Poirier, François Medjkane, Julie Brunelle, Véronique Drouineaud, Ouriel Rosenblum, Nouria Gründler, François Ansermet, Bruno Falissard, David Cohen, Agnès Condat

**Affiliations:** ^1^Clinique Dupré, Fondation Santé des Etudiants de France, Sceaux, France; ^2^Service de Psychiatrie de l'Enfant et de l'Adolescent, Hôpital Pitié-Salpêtrière, Paris, France; ^3^Service de Psychiatrie de l’enfant et de l’adolescent, Département de l’enfant et de l’adolescent, Hôpitaux Universitaires de Genève, Geneva, Switzerland; ^4^Centre de recherche en Epidémiologie et Santé des Populations, INSERM, U1018, Paris, France; ^5^Institut des Systèmes Intelligents et de Robotiques, Université Pierre et Marie Curie, Paris, France; ^6^CESP INSERM 1018, ED3C, Université Paris Descartes, Paris, France; ^7^CECOS Paris Cochin, Hôpital Cochin, Paris, France

**Keywords:** transgender, reproductive technologies, adolescent, psychological well-being, child

## Abstract

**Background:**

Today, individuals and couples with fertility issues can use advances in biomedical technologies to conceive. Transgender persons also benefit from these advances and can not only actualize their self-identified gender identities but also experience parenthood. These strategies for persons to self-actualize and to access parenthood have improved the condition of transgender persons. However, some may question the welfare of the offspring because such transfamily forms are often confusing to many. The sparse research on the psychological well-being of children of transgender people is reassuring. However, the limited empirical research justifies more studies to be conducted with an evidence-based methodology to assess whether these new methods of parenting have any adverse impact on children.

**Aims:**

The current report details the protocol we built to compare cognitive development, mental health, gender identity, quality of life, and family dynamics in children of transgender fathers and donor sperm insemination (DSI) and two control groups matched for age and gende typically developing (TD) children and children from cisgender parents and DSI.

**Hypothesis:**

To calculate sample sizes, we hypothesize no significant difference between groups.

**Subjects and Methods:**

Since 2008, married couples that include a transgender father have been able to access DSI and have started conceiving children in France. They are always invited to participate in research to assess their children's well-being. To date, the cohort includes 53 children in 37 families. We propose to carry out a cross-sectional comparative study exploring cognitive development with the Brunet–Lézine Psychomotor Development Scale or Wechsler's Intelligence Scales according to age; mental health with the Child Behaviour Checklist; gender identity with the Gender Identity Interview for Children; quality of life with the KIDSCREEN and the Adolescent Coping Questionnaire; and family dynamics with the Parental Bonding Instrument, the Inventory of Parent and Peer Attachment, the Five-Minute Speech Sample, and Corman's Family Drawing Test. To assess possible subtle differences between children's family drawings, we will use a generalization of the “lady-tasting-tea” procedure to link qualitative and quantitative approaches in psychiatric research. Twenty raters [four child and family psychoanalysts (CHILDPSY), four adult psychiatrists (ADUPSY), four biologists working in assistive reproduction technology (BIOL), four endocrinologists working with transgender individuals (ENDOC), and four students (STUD)] will be randomly shown the drawings and asked to blindly classify them using a Likert scale according to whether the child has a transgender father.

**Statistical Analysis:**

After testing normality, comparisons between the three groups will be performed with appropriate statistical tests (Kruskal–Wallis, ANOVA, Chi2 or Fisher's exact test). For the “lady-tasting-tea” procedure, we will use a permutation test.

**Ethics:**

The study protocol has been approved by the CERES (*Comité d'Ethique de Recherche en Santé*) of Paris 5 University. Registration number is 2015/31.

## Strengths and Limitations of the Protocol

- It is the first evidence-based protocol to assess the psychological well-being of the offspring of transgender persons.
- A number of outcomes will be used to comprehensively assess the development of the offspring of transgender persons.
- When the study is completed, the results should extend our understanding of the development of children born to anonymous cisdonors, as we will include two control groups.
- An original method will be applied to capture eventual subtle differences in the family dynamics through Corman's Family Drawing Test.
- The limitations include (i) the mean age of the children, knowing that only one-third will have reached adolescence and none will have reached adulthood; (ii) the heterogeneity of children's ages, meaning that some assessments will not be completed with all participants; (iii) the sample size, meaning that minor differences will be difficult to catch due to lack of statistical power; and (iv) the lack of exact matching expected for the children from cisgender parents and DSI. Indeed, cisgender parents who conceived with DSI do not usually inform their children; also, this group has been proposed to be contacted for developmental research more recently, meaning that we expect this group to be younger.

## Introduction

Today, biomedical technological advances have allowed individuals and couples with fertility issues to conceive (1). Medical advances in assisted reproduction technology have also created ways for human couples to access parenting. Additionally, medical progress in the field of gender dysphoria (GD) has allowed subjects to change their physical appearance and adopt the gender that they perceive to match their psychological identity. Consequently, transgender persons can not only actualize their self-identified gender identities, they can also benefit from advances in medically assisted reproduction (MAR) and experience parenthood. These new strategies for persons to self-actualize and to experience parenthood have improved conditions for transgender persons (2). However, they have raised several issues. In many countries, these advances have led to not only lively societal debates but also real ethical issues, the ultimate challenge being the well-being of the unborn child.

To enable transpersons to become parents outside the context of adoption, biomedical advances have introduced ways to conceive a child. In doing so, they have also introduced new changes in the ancestral logic of conceiving. The traditional bounds of gender identity, sexuality, conception, gestation, procreation, and filiation are deeply challenged (3). If the spread of contraception over the last 50 years has caused an effective separation between sexuality and procreation, the current disruptions in conservative thinking are going much further. Accessing parenthood with new MAR methods implies distinguishing the social father, the social mother, the genetic mother (oocyte donor), the genetic father (sperm donor), and the gestational mother (2). These advances are confusing to many and have led to lively societal debates opposing 2 different views: bio-catastrophists, on the one hand, and bio-prophets, on the other hand (4). The former think that science serves as a driving force to bring about apocalyptic times. It contributes to the destruction of norms and traditional modes and understandings of the meaning of life, with severe consequences for society and, ultimately, the resulting end of the human species. The latter believe that science offers the promise of a paradisiac future: a new redemptive era with a pure incorporeal spirit emerging from thinking machines.

In addition to provoking societal debates, these ways to self-actualize and conceive a child prompt several ethical issues, including first, puberty suppression and early school transition in adolescents with GD, which are largely allowed in several countries (e.g., Netherlands) but are not yet common in others (e.g., France) (5). Second, when a transgender person decides to enter into gender transition, the preservation of fertility raises specific issues for this individual, although this question is also relevant to other medical situations such as chemotherapy (6). Third, MAR technologies directed at transgender people also have specific consequences to be discussed: e.g., is it legitimate to have oocyte donors and gestational mothers tackling risks inherent to ovarian stimulation and to pregnancy, delivery, and eventually their own families to provide infertile parents with access to parenthood? Finally, some may question the welfare of the offspring because they think such transparenthood may be confusing for children. The current protocol aims to address this question.

The literature on the psychological well-being of transgender people's children is sparse. However, it does not support the idea that transparenthood negatively impacts children's development. The four studies in which children were born before their parent's transition are summarized in **Table 1** None of the children developed gender identity problems, but some individuals had psychiatric problems. Although Green published an anecdotal report of three cross-generational GD families (11), there is no known causative link in these cases, and compared to the total population of transgender people accessing parenthood, the cross-generational families are few in number (9).

**Table 1:** Summary of the literature studying the psychological well-being of transgender people's children

	N	Age	Gender identity problem
Green et al. (7)	37	5-16	None
Green et al. (8)	36	3-20	None
Freedman et al. (9)	18	3-15	None
White et al. (10)	55	8-35	None

However, the best situation to study the eventual impact of transgender parenthood is to explore children who are conceived by transgender people after their transition. In that case, children do not have to adapt to a new parental identity and are less confronted by socially aversive reactions. To our knowledge, only one study has been issued so far on this topic: 52 children born between 2000 and 2015, from donor sperm insemination (DSI) for couples with a transgender man (female to male), have been followed every two years. The qualitative results show that the children have normal development without any major psychological morbidity or GD (12). Most of the children participants knew that they were born by third-party MAR and that their fathers were born as females. However, the study did not include quantitative standardized evaluations and control groups; also, the assessments of the children were not blind.

In continuity with this last study, we propose to carry out a two-year cross-sectional comparative study of this cohort, now including 53 children in 37 families, using standardized quantitative assessments. We aim to compare cognitive development, mental health, gender identity, quality of life and family dynamics in children from transgender fathers and DSI and in two control groups matched for age, gender, and family status (in-couple vs separated): typically developing (TD) children and children from cisgender parents and DSI. To take into account the specificities that can be linked to the DSI as a mode of conception, we felt it was essential to have a control group of children born by conventional DSI as well as a control group of children who were conceived naturally. Additionally, to avoid questions about the results generated from the study being influenced by research staff opinions or beliefs, we will ensure that several comparisons of the children's status will be performed blind.

## Hypothesis

Our hypothesis is that the psychoaffective development of children born by DSI whose father is a transgender man does not significantly differ from that of children born with conventional DSIs or conceived by sex between cisgender parents. We aim to compare psychoaffective development in children conceived by DSI whose father is a transgender man (female to male) by conventional DSI and conceived by sex. To perform this protocol, we will collect objective data regarding cognitive development, mental health, gender identity, life quality, and family dynamics of children with an uncommon parenthood configuration, namely, transgender parenthood. We believe that this research will also improve healthcare for transgender couples and their children in a society where access to healthcare is potentially limited and remains difficult for this population (13). To calculate sample sizes, we hypothesize no significant difference between groups.

To explore more subtle differences between children born from DSI with a father of transsexual origin (female to male) and children born from natural conception, the protocol will include an experimental procedure previously developed to explore how traumatic experience could be predicted without explicit information through participants' responses to an experimental task using a permutation test (14, 15). In Cohen et al.'s study, the authors aimed to assess whether specific abilities enhance the recognition of implicit knowledge related to individual self-experience (15). To do so, they collected a series of videos from healthy adults who had experienced a sibling's cancer during childhood and matched controls. Subjects and controls were asked to give a 5-minute spontaneous free-associating speech following specific instructions created to activate a buffer zone between fantasy and reality. Then, several groups of raters were randomly shown the videos and asked to blindly guess which speaker had a sibling with cancer using a Likert scale. Using a permutation test, they found that only psychoanalysts were able to recognize, above levels of chance, healthy adults who had experienced sibling cancer during childhood without explicit knowledge of this history. In contrast, medical students, oncologists, cognitive behavioral therapists and individuals who had the same history of a sibling's cancer were unable to do so. In our protocol to explore children's family experiences, we will ask all participating children to provide a family drawing that will be subsequently blindly shown and classified by raters to guess the children's group. We hypothesize that no group of raters will guess the children's group above the level of chance.

## Methods and Analysis

### Design and Recruitment of the Participants

The study design is a monocentric cross-sectional comparative protocol study over 2 years. Of note, the CECOS-Cochin center is unique; itis authorized to treat couples from all over France. We will establish three groups of children of the same size, matched by age and sex. Two groups will be recruited from couples who have already met at the CECOS-Cochin and have at least one child born by DSI. The first group of children consists of those born by DSI where the father is transgender (female to male). Fifty-three children (from the 37 families) were recruited, which is considered to be the maximum number of participants.

The second group of children consists of those born by conventional DSI. Approximately 75 couples have a progressive pregnancy by DSI at CECOS-Cochin annually. It is common for these couples to return with a second or third request for insemination. These couples will be invited to participate in the study. This proposal may be formulated during a consultation requested by the couple for whatever reason, if the couple already has a child. Another way to contact other couples may be by telephone, provided that they have given their written consent to be contacted again by the CECOS at the time they enrolled to receive the DSI.

The third group includes controls born by sex between cisgender parents. They will be recruited by announcement in the meetings of the departments involving the families of the professionals who will agree. Our goal is to conduct the protocol with at least 45 subjects (1 subject = 1 child) in each of our three cohorts (see *Data Processing, Statistics, and Power Calculation* below). Inclusion criteria include a target population of girls and boys aged between 18 months and 15 years of age, born by DSI or naturally conceived for the control group, who agreed to participate in a one-day evaluation with the consent of their parents. Exclusion criteria are (i) a poor understanding of written and/or spoken French, which would not allow participants to correctly fill in the questionnaire forms or pass the standardized interviews, and (ii) a refusal from at least one parent to sign the consent form.

### Measures and Procedure

Each child and family will undergo a thorough evaluation within the Department of Child and Adolescent Psychiatry at the Pitié-Salpêtrière Hospital in Paris. The department is well suited to a 1-day welcoming of children for these assessments. The protocol explores five axes (**Table 2**): (i) cognitive development using an adapted rating according to age (Brunet–Lézine psychomotor development scale(16) from 2 to 30 months, the WPPSI-III (17) from 30 months to 7 years old, and the WISC-4 (18) from 7 years old and up); (ii) mental health using Achenbach's Child Behaviour Checklist (CBCL) (19); (iii) gender identity appreciation using the Gender Identity Interview for Children (GIIC) (20); (iv) quality of life using KIDSCREEN 52 (21) and the Adolescent Coping Scale (ACS) (22, 23); and (v) family dynamics and interaction using the Parental Bonding Instrument (PBI) (24), the Inventory of Parent and Peer Attachment (IPPA) (25), the Five-Minute Speech Sample (FMSS) (26), and the Corman's Family Drawing Test (27).

**Table 2:** Study measures.

Name of the instrument	Characteristics
***Cognitive development***	
Brunet–Lézine Developmental Examination (16)	It estimates a developmental quotient (DQ) based upon normative data available for 2-year-old French toddlers.
WPPSI (17)	It is a standardized developmental test for preschool-age children to measure intelligence skills
WISC-4 (18)	It is a standardized developmental test for school-age children to measure intelligence skills
***Mental health***	
CBCL (19)	It assesses global psychopathology. It is a parent-report measure designed to record the behaviors of children. Each item describes a specific behavior, and the parent is asked to rate its frequency on a three-point Likert scale. The scoring gives, among others, three main scores (Internalizing, Externalizing, Total Problems): a T-score of 63 and above is considered clinically significant; values between 60 and 63 identify a borderline clinical range; and values under 60 are considered non-clinical.
***Gender identity***	
Gender Identity Interview for Children (20)	It assesses affective and cognitive gender confusion within the child.
***Quality of life***	
KIDSCREEN 52 (21)	It assesses the child's global quality of life. It presents as a questionnaire for children and young people and measures 10 health-related quality of life dimensions: Physical- (5 items), Psychological Well-being (6 items), Moods and Emotions (7 items), Self-Perception (5 items), Autonomy (5 items), Parent Relations and Home Life (6 items), Social Support and Peers (6 items), School Environment (6 items), Social Acceptance (Bullying) (3 items), and Financial Resources (3 items).
Adolescent Coping Scale (22, 23)	It assesses how adolescents cope with a situation or resolve a problem by scoring three main factors: (a) productive coping); (b) non-productive coping; and (c) reference to others.
***Family dynamic and interaction***	
Parental Bonding Instrument (24)	It measures fundamental parental styles as perceived by the child. The measure is ‘retrospective', meaning that young adults (over 16 years) complete the measure of how they remember their parents during their first 16 years. The measure is to be completed for both mothers and fathers separately. There are 25 item questions, including 12 ‘care' items and 13 ‘overprotection' items.
Inventory of Parent and Peer Attachment (25)	It measures various qualities of the child's relationships with parents and peers, including trust, quality of communication, and feelings of anger and alienation. It contains three sub-questionnaires, one concerning the mother, one concerning the father and one concerning peers.
Five-Minute Speech Sample (26)	It assesses expressed emotions within the family. It measures levels of criticism, emotional over-involvement, warmth and positive remarks made by a relative toward the child.
Corman's Family Drawing Test (27)	It assesses the child's perception of the family relationship. After the child has finished the drawing, she or he is asked some questions to determine her or his feelings and thoughts and gain a better understanding of the drawing.

WPPSI, Wechsler Preschool and Primary Scale of Intelligence; WISC-4, Wechsler Intelligence Scale for Children (4^th^ edition); CBCL, Child Behaviour Checklist.

The protocol will take place on a single day, in the psychiatry department of the Pitié-Salpêtrière or at home if the parents wish. During cognitive assessment of the children, each parent (both mothers and fathers) will fill out the Standardized Sociodemographic Questionnaire, which collects information about the child and his or her living environment and education, and the CBCL parent questionnaire. Then, the FMSS will be made with both parents by one of our trained clinicians. After the lunch break, parents will have a semi-structured interview, conducted by a psychiatrist, who will focus on their parenting experience. Meanwhile, a clinical psychologist will evaluate the mental health of the child using the CBCL child questionnaire if his or her age allows it and will ensure the passing of the GIIC. Self-administered questionnaires will then be offered to the child according to his or her age: the KIDSCREEN 52 for the evaluation of her or his quality of life, the Coping Scale for Adolescents, the PBI and the IPPA. Finally, the child will be asked to provide a family drawing. At the end of the day, and after an exchange between the involved clinicians, one of the psychiatrists will offer feedback to the child and his family regarding the immediate results of the clinical evaluation, and advice can be given if needed.

To maintain blind assessments, the psychologist who will follow how children and parents fill in the self-report questionnaires will remain blind to the children's group. Additionally, the cognitive assessment and the experimental procedure (the family drawing) will be performed in a blinded manner. Finally, we will also run all ratings and statistical analyses in a blinded manner. However, the parents' semi-structured interviews with the psychiatrist will not be blind, as some questions focus on parenting.

### Data Processing, Statistics, and Power Calculation

As requested by French regulations, all data will be processed anonymously and confidentially. Data will be identified only by a code number, and correspondence between this code and the participants' name/surname can only be established through a private list kept separately in another office. We will use the Pitié-Salpêtrière child psychiatry computerized database for the processing of these data (CNIL declaration n° 1303778). The data will be directly integrated into computer software on a laptop regularly entrusted for analysis to the statistician engineer of the Department of the Child and Adolescent Psychiatry of the Pitié-Salpêtrière hospital.

Taking into account the purpose of the study, statistics will be essentially descriptive: frequencies and percentages for the qualitative variables, means, standard deviations, minimum, maximum, and median for the quantitative variables. In addition, the norms in the general population (or even by age category/sex if available) of the different instruments used will make it possible to calculate for each patient a Z-score according to the following formula: Z = (X − μ)/σ, where μ and σ represent the mean and standard deviation in the general population of the variable X measured by the instrument. This Z-score reflects the distance of an individual from an average person. This should estimate finely how many patients have scores significantly far from the average. For example, with a 5% threshold, a Z-score greater than 1.96 in absolute value corresponds to an individual significantly different from the norm.

In addition to an analysis based on the standard available to the general population, the results will be compared to those obtained for children in the control groups. As we hypothesize that the psychoaffective development of children born by DSI whose father is a transgender man will not significantly differ from that of other groups, we need to ensure that the number of individuals included is high enough to ensure that if we had no difference between groups, the statistical power is sufficient. The minimum size of the sample was calculated to be able to show with an alpha error probability of 5% and a statistical power of 80% a significant difference between two groups on the CBCL, one of our main objectives. We used the Multicultural Supplement to the Manual for the ASEBA School-Age Forms & Profiles (28) baseline data that present means and standard deviations of the French population for each scale. We calculated for each scale the sample size needed using the statistical program R, version 3.3.1 (R Foundation for Statistical Computing) (29) with the formula n.for.2means (m1, m2, sd1, sd2, ratio, alpha, power). The minimum size ranged between 3 and 24 per group according to CBCL subscales. Only the CBCL total score required a minimum size of 38 per group. Therefore, to take into account missing data, we aimed to include approximately 45 patients in each group.

To explore more subtle differences between children born by DSI and a father of transsexual origin (female to male) and children born from natural conception, the protocol will include an experimental procedure that was previously developed to explore how traumatic experience could be predicted without explicit information, through participants' responses to an experimental task using a permutation test (14, 15).

Here, the task we will propose to the children is the family drawing (27). We hypothesize that children born by DSI and transsexual fathers would be more likely during the family drawing test to use atypical representations (e.g., of men/fathers, of sexual indices). Raters with diverse experiences would eventually be in position to guess the children's group by viewing the drawings. To explore which experiences in raters may be helpful, the family drawings will be analyzed by 20 raters (4 child and family psychoanalysts (CHILDPSY), 4 adult psychiatrists (ADUPSY), 4 biologists working in assistive reproduction technology (BIOL), 4 endocrinologists working with transgender individuals (ENDOC) and 4 students (STUD)). They will be randomly shown the drawings and asked to blindly classify them according to whether the child had a transgender father using a four-level Likert scale. Differences between children's family drawings will be evaluated with a generalization of the “lady-tasting-tea” procedure to link qualitative and quantitative approaches in psychiatric research (14). Qualitative interviews will be conducted with the contributors if differences are found. Given that the family drawing is possible with children aged 4 years and older, we expect to recruit between 15 and 20 children per group for this task. **Table 3** enables power and sample size calculation.

**Table 3:** Power and sample size calculation for the family drawing experiment [extracted from Falissard et al. (14)]

	n = 2 × 15	n = 2 × 20
k = 1	0.24 0.5 0.82	0.28 0.62 0.9
k = 2	0.34 0.74 0.97	0.40 0.85 0.99
k = 3	0.43 0.87 >0.99	0.51 0.95 >0.99
k = 5	0.58 0.97 >0.99	0.67 0.99 >0.99

Statistical power of the experimental procedure for three alternative hypotheses (sensitivity and specificity of raters equal to 0.6 (above), 0.7 (medium) 0.8 (below)) for n drawings scored by k raters (two-sided test with a type-1 error equal to 0.05).

Example: for n = 2 × 20 drawings scored by three raters, the statistical power is equal to 0.95 for the alternative hypothesis that the raters discriminate the two groups with a sensitivity and a specificity of 0.7.

## Discussion

As of today, we have no knowledge of an evidence-based study to assess whether new ways of parenting have any adverse impact on children. Nevertheless, such literature is essential, as physicians are currently confronted with transgender families on a regular basis. On the medical side, the results of this study, when completed, will provide a better understanding of the development of children born by anonymous transparents and will help increase knowledge about children born by DSI more generally. On the psychological level, the results will help to deepen our knowledge concerning the representation for the child of the father and the paternal function. The results could constitute support for reflection by ethics committees and doctors subjected to certain questions concerning the future of these children. This may also help general practitioners, child psychiatrists and psychologists take care of children born by these donations. The results of psychological assessments can be communicated to children and parents in accordance with the child's wish. If any problem is detected during the assessment of a child that could lead to identified care or require special precautions, parents will be informed. When appropriate, help by a trained psychiatrist or psychologist will be proposed to parents who wish to reveal to their child how they were conceived but are having difficulty doing this on their own.

### Ethics and Dissemination

The study protocol was approved by the CERES (*Comité d'Ethique de Recherche en Santé*) of Paris 5 University. Registration number is 2015/31. Informed and written consent will be obtained from parents and children for participation in the study. Appropriate information will be provided to parents and the children or adolescents according to their age, orally up to 11 years old and with a written document for parents and for children and adolescents aged 12 to 15 years old.

When parents did not give their child born by DSI information about how he or she was conceived, we will respect their wish and will not reveal it. Children and adolescents will therefore be informed of the general objectives of the study in which they will participate but not of their status among the three possible groups. The information will be given to them as follows: “*Children are most often conceived by their parents, but parents may not be able to have children without the help of doctors. This research tries to find out if the way a child has been conceived can have effects on his psychological and emotional development*.” However, if parents manifest the wish to use these interviews to reveal to the child their mode of conception, we will propose accompaniment.

We seek to publish the results of our study when completed for peer review in an academic journal. Findings will also be presented to researchers and clinicians at suitable conferences.

## Ethics Statement

The study protocol was approved by the CERES (Comité d'Ethique de Recherche en *en Santé*) of Paris 5 University. Registration number is 2015/31. Informed and written consent will be obtained from parents and children for participation in the study. Appropriate information will be provided to parents and the child or adolescent according to their age, orally up to 11 years old and with a written document for parents and for children and adolescents aged 12 to 15 years old.

## Author Contributions

AC, DC, VD, and BF designed the protocol. AC, CL, JW, NM, JB, FM, and VD are in charge of the recruitment and assessment of the participants. AC, DC, VD, NG, and FA discussed ethical aspects and made the regulation submissions. GM, DC, AC, and BF designed the statistical analyses. GM, CL, AC, and DC drafted the first version of the MS. All authors approved the final version of the MS.

## Funding

AC has received funding for this project from the Pfizer Foundation. The funder was not involved in the study design, collection, analysis, interpretation of data, the writing of this article or the decision to submit it for publication.

## Conflict of Interest

The authors declare that the research was conducted in the absence of any commercial or financial relationships that could be construed as a potential conflict of interest.
